# Secular Trend, Seasonal Variation, Epidemiological Pattern, and Outcome of Traumatic Head Injuries Due to Road Traffic Accidents in Aseer, Saudi Arabia

**DOI:** 10.3390/ijerph18126623

**Published:** 2021-06-20

**Authors:** Mubarak Ali Algahtany

**Affiliations:** Division of Neurosurgery, Department of Surgery, College of Medicine, King Khalid University, Abha 62512-2291, Saudi Arabia; mbalgahtany@kku.edu.sa

**Keywords:** road traffic accident, traumatic head injury, trend, seasonal variation, Saudi Arabia

## Abstract

Road traffic accidents (RTAs) are a leading cause of traumatic head injury (THI) and are regarded as a public health problem in Saudi Arabia. This hospital-based retrospective study aims to provide data on the frequency, type, and distribution of RTA-related THIs over the past decade; demonstrate their time trend and seasonality; and decipher age and sex differences in RTA-related THIs and their outcome. The results showed a decline in the number of RTA-related THIs between 2010 and 2019. The patients had a mean age of 26.16 ± 16.27 years, and the male-to-female ratio was 10.8:1. Head injury with multiple lesions was the most common diagnosis, followed by cerebral contusion and skull fracture (32.1%, 12.9%, and 11.2%, respectively). Subdural hematoma (SDH) and skull fracture were significantly more common in patients aged ≥60 years (standard residual > 1.96), and significantly less common in those aged ≤17 years (standard residual < 1.96), compared to other age groups. Males experienced significantly more SDHs than females (standard residual = −2.8, *p* = 0.029). The length of hospital stay was positively correlated with age (Spearman’s rho = 0.057, *p* = 0.046). No seasonal variation was found.

## 1. Introduction

Road traffic accident (RTA)-related injuries are a leading cause of morbidity and mortality worldwide [1]. RTAs mostly affect young males, ranking first among the causes of disability-adjusted life in the 10–49-year-old age group [2]. It must be noted that RTAs contribute 2.12% to the total global mortality; however, this figure increases to 16.54% in Saudi Arabia, where it is the leading cause of death in young adult males [3,4]. Among RTA-related injuries, traumatic head injury (THI) is considered severe, and RTA is the leading cause of THI-related deaths worldwide [5,6,7,8], reaching up to 41% in the Middle East and North Africa (MENA) regions [9]. Thus, it is clear that RTA-related THIs impose a substantial clinical, social, and economic burden on individuals and health-care systems globally.

Saudi Arabia has implemented aggressive measures over the past decade to address the problem of RTAs, including introducing stricter traffic regulations and ensuring their stringent enforcement. These changes peaked after the launching of Vision 2030 in April 2016. Though the reports from police department in Aseer province portray a decreasing trend in RTA since 2016, the effect on RTA-related injuries is a matter of further deliberation [10].

In order to assess the THI burden and deliver the highest quality medical care for trauma patients, data on time trends, seasonal variations, and epidemiological characteristics are critical. Previous research in other parts of the world has shown that seasonal increases in traffic accidents may be weather- or tourism-dependent, due to the temporary increase in population [11]. This is of particular concern in Aseer, as it is a tourist destination for the Arab world in the summer months [12]. However, there are no previous studies from Saudi Arabia on the influence of external factors, such as the seasonal effect or day of the week, on THI incidence, and no previous study has specifically addressed RTA-related THI in Saudi Arabia. Therefore, the primary objective of this study was to provide much needed information on the frequency, type, and distribution of RTA-related THI over a 10-year span, to demonstrate the time trends and seasonal variation. The secondary objective was to decipher age and sex differences among individuals with THI and their outcome. It is expected that quantifying these relationships would help identify the at-risk groups, leading to better planning and management.

## 2. Materials and Methods

### 2.1. Type of Study

This was a retrospective, record-based study.

### 2.2. Study Area

Aseer province, Southwest Saudi Arabia. The Aseer region is in the southwest of the country, with an area of 76,693 square kilometers (29,611 sq mi), and an estimated population of 2,308,329 people in 2019 [10]. As the tourism capital of the Gulf region, road traffic is increased manifold in the summer months. As the only tertiary hospital in the province, Aseer Central Hospital (ACH) typically handles the majority of THIs.

### 2.3. Study Sample and Technique

This study included 1235 cases of RTA-related THI admitted to ACH between 1 January 2010 and 31 December 2019. Collected variables included the sociodemographic characteristics, trauma details, hospital admission details, and in-hospital outcome. The data were complete for all variables of interest.

### 2.4. Statistical Analysis

After coding, analysis was conducted using the SPSS statistical package (version 23, IBM, Arnmonk, NY, USA). Frequencies and percentages are used to describe the number of cases over different time templates, as in year, month, season, and workday/weekend, and the time trends are represented as charts. Injuries were classified and described based on the type, region of involvement, and outcome in terms of intensive care unit (ICU) admission, hospital length of stay (LOS), and mortality. All the above variables were analyzed for differences between sexes and age groups using the Chi-square test. Standard residuals (SR) were used to study the strength of the difference between observed and expected values. Results were considered significant at 95% CI and *p* < 0.05.

## 3. Results

A total of 5830 RTA-related injuries were presented between January 2010 and December 2019 to the study center. Out of this, 1235 (21.1%) had RTA-related THI.

**Figure 1:**
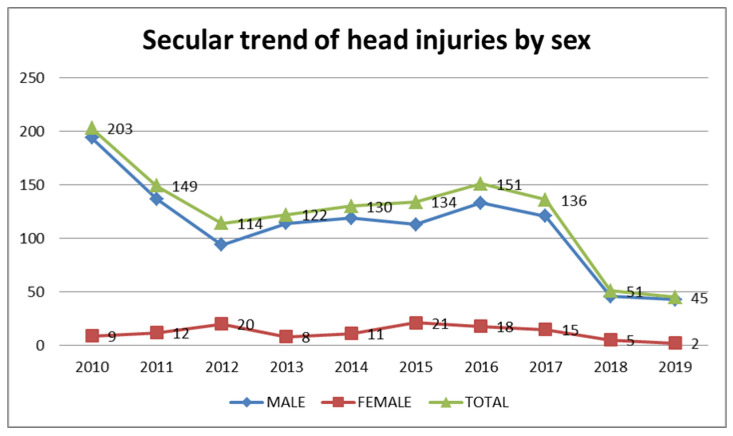
Secular trend of traumatic head injuries in Aseer, Saudi Arabia.

Table 1 and Figure 1 illustrate the decreasing trend of RTA-related THI in Aseer province, Saudi Arabia. There was an initial sharp fall between 2010 and 2012, after which there was a slight rise over the next four years. From 2016, the year in which Vision 2030 was launched, with the adoption of firm traffic regulations, there was a remarkable fall in the number of cases; however, the drop was not statistically significant.

The monthly distribution reveals a higher number of cases (37%) in the months of June, July, and August (the summer season), but this was not statistically significant compared to the other seasons (Table 1). There was also no difference between the number of cases admitted in the weekend vs. those admitted on the weekdays.

Analyzing the gender differences reveals an overwhelming preponderance of male patients (90.2%), with a male-to-female sex ratio of 10.8:1. The analysis of gender-related time trends revealed a significantly low proportion of female cases in 2010, but a high number of female cases in 2012 and 2015 (*p* = 0.004, standard residual value −2.4, 2.6, and 2.2, respectively). The month-wise comparison showed that female cases were significantly higher in August and lower in February (*p* = 0.017). Otherwise, there was no statistical difference between male and female patients according to season or day of the week.

Table 2 shows that the most common RTA-related THI diagnosis was head injury with multiple lesions (32.1%), followed by cerebral contusion (12.9%) and skull fracture (11.2%). Associated non-head injuries were present in more than half (52.6%) of the patients, with a tendency to be more prevalent in females. The majority (81.1%) of the cases were managed in the regular ward. The LOS in the hospital was divided into five groups, as shown in Table 2; most (38.6%) of the cases were hospitalized for less than a week, while less than 2% were hospitalized for more than six months. Although 81.4% of the patients were discharged home after improvement, the fatality rate was 14.7%. With regards to the gender-related pattern of injury, the incidence of subdural hematoma was significantly lower in females than in males (SR = −2.8, *p* = 0.029). The average LOS was 23.77 ± 50.97 days, while it was higher in males than in females (24.19 ± 52.91 vs. 19.92 ± 27.21 days, respectively), the difference was not statistically significant. There were no gender-specific differences in ICU admission or mortality (Table 2).

Table 3 presents the age differences of patients with RTA-related THI. The mean age of the patients was 26.16 ± 16.27 years (range, 2 months–85 years). The mean age of the males was 26.14 ± 15.86, and that of the females was 26.36 ± 19.74 years. To analyze the differences over different age spans, we divided age into four groups, as shown in Table 3. Age had no effect on the injuries received at different times of the year (months, season) or time of the week (weekend). Although there was a drop in the number of cases among all age groups after the launching of Vision 2030 in 2016, it did not have statistical significance.

Table 4 presents the pattern of injuries and their outcome in the different age groups. There were statistically significant differences in the type of head injury by age group. The 0–17-year-old age group had significantly fewer occurrences of subdural hematoma and skull fracture (standard residual < 1.96), and significantly more occurrences of subarachnoid hemorrhage (standard residual > 1.96) than that found in the other age groups. In contrast, the ≥60-year-old age group had significantly more occurrences of subdural hematoma and skull fracture (standard residual > 1.96), and significantly fewer instances of EDH (standard residual < 1.96) than that found in the other age groups. No significant differences were observed in the need for ICU admission and the outcome between the age groups. When age was grouped, there were no significant differences between the LOS in the different groups; however, when analyzed as a continuous variable, age had a significant positive correlation with LOS (Spearman’s rho = 0.057, *p* = 0.046).

## 4. Discussion

RTAs and their resulting THIs represent a major public health problem. The present study addresses the demographics of THIs caused by RTAs in Aseer province, Saudi Arabia. Our aim was to study the time trends and pattern of THI in the region to help set the course for future action in head trauma prevention programs, and to guide its management. A total of 1235 patients with RTA-related head injuries presented between January 2010 and December 2019, representing 21.3% of all RTA-related injuries. The most encouraging observation in this study was the steady decline in cases of RTA-related THI over the decade. The number of cases per year fell from 203 in 2010 to 45 in 2019. The fall is remarkable after 2016. Saudi Arabia has recently adopted strict traffic regulations, synchronized with Vision 2030, which launched in April 2016. These include regulations on speeding, seat belt wearing, and mobile use while driving, which are strictly enforced by the most advanced traffic management technologies [13]. Evidence from previous studies has established a significant decline in RTAs after the implementation of the camera ticketing system [13] and Vision 2030 in the KSA [14]. Recently published data from the traffic department reveals a 22% decline in traffic accidents between 2016 and 2019. The total number of RTA dropped from 32,684 in 2016 to 25,342 in 2019 [10]. Our study is the first from Saudi Arabia to show consistency between police-reported data on RTA trend and RTA-related THI trend from health registration data [15]. In Japan and Russia, decreasing speed limits resulted in a favorable decrease in RTAs and their associated mortality rate [16,17]. We can interpret the results of this study in a similar way; however, we cannot state conclusively about the exact factors leading to the decline in cases. Although there is a huge drop in the number of RTA-related THIs after the 2016 announcement of Vision 2030, this decrease did not reach statistical significance, and therefore does not support the assumption that the change in regulations alone is the reason for the decrease in the number of cases. It is likely that other important factors, such as urbanization, changes in the types of cars and car safety measures, and improved road conditions, play a role in the trend observed in this study. Further collaborative research with other departments, including the road traffic department, is needed to study the factors affecting the change in the number of accidents and resulting injuries.

Although more males are involved in road traffic injuries worldwide, this divide is more pronounced in the Middle East region [18]. In our study, there was an overwhelming preponderance of male patients, consistent with the results of other studies from Saudi Arabia [19,20]. The reason for this difference is obvious, as there are more men on Saudi roads than women. However, in the past few years, more women have been allowed to drive and participate in work outside the home. It would be interesting to study future trends of sex differences in accidents and injuries in the region.

Studies have independently reported seasonal, weather, and temporal variations in traffic accident-related injuries, with reports of higher incidences of accidents during summer, on days with precipitation, on particular days of the week, and at specific times of the year [21,22,23]. This is the first study from Saudi Arabia on the seasonal variation in RTA-related THI. We found no relation between RTA-related THI and the day of the week, month, or season.

In this study, we went one step further to find the relationship between patients of specific age and gender groups presenting with RTA-related THI and the above-mentioned variations. A detailed analysis of time trends revealed differences in the incidence of female cases in specific years; however, these differences are difficult to explain, especially as these were prior to 2018, the year in which females were legally permitted to drive in the Kingdom. The number of cases dropped after 2016 in both males and females, and no significant difference was observed between the two groups.

Analysis of the type of injuries revealed head injury with multiple lesions, cerebral contusion, and skull fracture to be the most common diagnoses. Fewer than one in five cases were admitted to the intensive care unit. These statistics are lower than those reported in Riyadh, which is a large metropolitan city with heavy and fast-moving traffic [24]. Similar to reports in other studies, the majority of patients in this study (38.6%) stayed in the hospital for less than a week, while less than 2% were hospitalized for more than six months [25]. The findings of more skull fractures and SDH in senior adults compared to children could be explained by the increase in bone fragility and brain atrophy found in older adults.

According to the Global Burden of Disease study, the mortality rate of RTAs was 2.3% in 2017 [26]. Various factors affect the incidence and mortality of traffic-related injuries, including vehicle design, speed control, road infrastructure, and traffic law enforcement. However, the site and severity of injury remain the most important factors in determining the outcomes of road traffic injuries. In this study we focused on head injuries, which are known to have a higher fatality than other injuries. The fatality rate in our patients was 14.7%, with no significant difference between the male and female patients. This figure lies between the 5.9% and 30% mortality rates reported by studies from other locations in the region [6,19]. The lack of difference in mortality between male and female patients, despite the significance of SDH in males, could be due to other confounding factors, such as an increase in associated non-THI in females, as shown in this study.

The mean age of the patients was 26.16 ± 6.27 years, which is younger than that reported in the capital city of Saudi Arabia, where the mean age was 32.6 years (±14.7) [19]. This could be related to the relatively younger population in Aseer province in comparison to Riyadh [10]. We found no association between ages and the times of the year (months, season), or time of the week (weekend) that people experienced accidents. Though more patients presented in the summer months, which corresponds with the holiday and tourism season in the region, this trend was not significant or associated with any age or sex group. However, we are limited by the lack of data on the local vs. non-local nature of the victims to relate the number of accidents to tourism. Interestingly, the foggy winter season in Aseer was not associated with an increase in RTA-related THI. This is supported by a study that found that bad weather conditions in the wintertime do not lead to an increased number of accidents [27]. These findings have positive implications as they suggest that health services are not overburdened on vacation or weekends by RTA-related head injuries.

We found a significant positive correlation between age and LOS, consistent with findings by other authors [28]. This implies that while RTA-related THI tends to happen more in the younger population, the presence of this injury in the older population consumes more health resources.

## 5. Conclusions

This study provided unique insights into the trends, patterns, age and gender differences in the presentation and outcomes of RTA-related THI. The remarkable decrease in the number of cases over the past decade enforces that progress has been made to prevent RTA and related head injuries. Skull fractures and SDH affect older persons more than children. Though RTA-related THI is more common in the young, senior adults tended to have longer LOS and use more health resources. Thus, based on the results from this study, preventive measures directed toward at-risk groups could further decrease the incidence of RTA-related THI. Our study supports that the strict implementation of stringent traffic regulations could play a role in reducing the health-related burden of RTA.

### Strength and Limitations

This is the first and largest study to focus upon the time trend, seasonal variations, and age-sex differences of RTA-related head injuries in Saudi Arabia. However, there are some limitations of the study. Firstly, it is a hospital-based study; thus, the information may not be a true reflection of the population. Secondly, it included only admitted patients; therefore, non-admitted minor head injuries or those who died before reaching the hospital were not captured. Moreover, the topography and climate of the Aseer province is quintessentially different from the rest of Saudi Arabia; hence, the results cannot be generalized to the whole of the kingdom. Nevertheless, it provides valuable data on a vital health problem that has not been previously addressed in such a manner.

## Figures and Tables

**Table 1:** Time trends and seasonal variation of traumatic head injury cases by gender (*n* = 1235).

Variable	Male *n* = 1114 (%)	Female *n* = 121 (%)	Total *n* = 1235 (%)	OR [CI 95%]	*p* Value
**Year**					
2010 * standard residual	194 (17.4)	9 (7.4) −2.4	203 (16.4)	---------	0.004
2011	137 (12.3)	12 (9.9)	149 (12.1)		
2012 * standard residual	94 (8.4)	20 (16.5) 2.6	114 (9.2)		
2013	114 (10.2)	8 (6.6)	122 (9.9)		
2014	119 (10.7)	11 (9.1)	130 (10.5)		
2015 * standard residual	113 (10.1)	21 (17.4) 2.2	134 (10.9)		
2016	133 (11.9)	18 (14.9)	151 (12.2)		
2017	121 (10.9)	15 (12.4)	136 (11.0)		
2018	46 (4.1)	5 (4.1)	51 (4.1)		
2019	43 (3.9)	2 (1.7)	45 (3.6)		
**Month**					
January	94 (8.4)	6 (5)	100 (8.1)		0.017
February * standard residual	87 (7.8)	3 (2.5) * −2	90 (7.3)		
March	98 (8.8)	6 (5)	104 (8.4)		
April	78 (7.0)	13 (10.7)	91 (7.4)		
May	79 (7.1)	14 (11.6)	93 (7.5)		
June	108 (9.7)	7 (5.8)	115 (9.3)		
July	112 (10.1)	11 (9.1)	123 (10.0)		
August * standard residual	109 (9.8)	21 (17.4) * 2.3	130 (10.5)		
September	97 (8.7)	8 (6.6)	105 (8.5)		
October	89 (8.0)	13 (10.7)	102 (8.3)		
November	94 (8.4)	13 (10.7)	107 (8.7)		
December	69 (6.2)	6 (5)	75 (6.1)		
**Season**				-----------	0.071
Winter	250 (22.4)	15 (12.4)	265 (21.5)		
Spring	176 (15.8)	19 (15.7)	195 (15.8)		
Summer	408 (36.6)	53 (43.8)	461 (37.3)		
Autumn	280 (25.1)	34 (28.1)	314 (25.4)		
**Day of week**					
Weekday	795 (71.4)	80 (66.1)	875 (70.9)	0.78 [0.52,1.16]	0.228
Weekend	319 (28.6)	41 (33.9)	360 (29.1)		
**Vision 2030**					
Before	771 (69.2)	81 (66.9)	852 (69)	0.901 [0.604,1.34]	0.608
After	343 (30.8)	40 (33.1)	383 (31)		

*: Denotes that significant difference exist.

**Table 2:** Pattern of head injuries and its outcomes by gender (*n* = 1235).

Variable	Male *n* (%)	Female *n* (%)	Total *n* (%)	OR [CI 95%]	*p* Value
**Head injury**					
Multiple lesions	360 (32.3)	37 (30.6)	397 (32.1)	---------	0.029
Concussion	94 (8.4)	14 (11.6)	108 (8.7)		
Contusion	146 (13.1)	14 (11.6)	160 (12.9)		
SDH * Standard residual	116 (10.4)	2 (1.7) * −2.8	118 (9.6)		
EDH	98 (8.8)	10 (8.3)	108 (8.7)		
SAH	36 (3.2)	5 (4.1)	41 (3.3)		
Skull fracture	118 (10.6)	20 (16.5)	138 (11.2)		
Superficial injury	36 (3.2)	6 (5)	42 (3.4)		
Unspecified injury	75 (6.7)	12 (9.9)	87 (7.0)		
Other (DAI, IVH, ICH)	35 (3.1)	1 (0.8)	36 (2.9)		
**Associated injury**					
No	534 (47.9)	51 (42.1)	585 (47.4)	0.79 [0.541,1.157]	0.226
Yes	580 (52.1)	70 (57.9)	650 (52.6)		
**ICU admission**					
No	902 (81)	100 (82.6)	1002 (81.1)	1.11 [0.683,1.834]	0.651
Yes	212 (19)	21 (17.4)	233 (18.9)		
**LOS**				------	0.483
Less than 1 week	433 (38.9)	44 (36.4)	477 (38.6)		
1–2 weeks	281 (25.2)	36 (29.8)	317 (25.7)		
15–30 days	188 (16.9)	19 (15.7)	207 (16.8)		
30–180 days	191 (17.1)	22 (18.2)	213 (17.2)		
>180 days	21 (1.9)	0 (0)	21 (1.7)		
**Outcome**				------	0.674
Discharge to home	910 (81.7)	95 (78.5)	1005 (81.4)		
Transfer	43 (3.9)	6 (5.0)	49 (4.0)		
Death	161 (14.5)	20 (16.5)	181 (14.7)		

*: Denotes that significant difference exist. EDH: epidural hematoma. SDH: subdural hematoma. SAH: subarachnoid hemorrhage. ICH: intracerebral hemorrhage. DAI: diffuse axonal injury. IVH: intraventricular haemorrhage. ICU: intensive care unit. LOS: length of stay.

**Table 3:** Time trends and seasonal variation in head injury cases by age (*n* = 1235).

Variable	0–17 Years *n* (%)	18–34 Years *n* (%)	35–59 Years *n* (%)	60 Years and above *n* (%)	Total *n* (%)	*p* Value
**Year**						
2010	49 (14.6)	124 (18.6)	18 (12.9)	12 (13.0)	203 (16.4)	0.499
2011	39 (11.6)	87 (13.0)	16 (11.5)	7 (7.6)	149 (12.1)	
2012	39 (11.6)	50 (7.5)	12 (8.6)	13 (14.1)	114 (9.2)	
2013	36 (10.7)	64 (9.6)	12 (8.6)	10 (10.9)	122 (9.9)	
2014	37 (11.0)	64 (9.6)	18 (12.9)	11 (12.0)	130 (10.5)	
2015	41 (12.2)	69 (10.3)	15 (10.8)	9 (9.8)	134 (10.9)	
2016	40 (11.9)	76 (11.4)	23 (16.5)	12 (13.0)	151 (12.2)	
2017	35 (10.4)	78 (11.7)	11 (7.9)	12 (13.0)	136 (11.0)	
2018	12 (3.6)	30 (4.5)	5 (3.6)	4 (4.3)	51 (4.1)	
2019	8 (2.4)	26 (3.9)	9 (6.5)	2 (2.2)	45 (3.6)	
**Month**						0.402
January	26 (7.7)	62 (9.3)	8 (5.8)	4 (4.3)	100 (8.1)	
February	23 (6.8)	52 (7.8)	9 (6.5)	6 (6.5)	90 (7.3)	
March	22 (6.5)	64 (9.6)	13 (9.4)	5 (5.4)	104 (8.4)	
April	29 (8.6)	48 (7.2)	7 (5.0)	7 (7.6)	91 (7.4)	
May	30 (8.9)	42 (6.3)	10 (7.2)	11 (12.0)	93 (7.5)	
June	31 (9.2)	58 (8.7)	15 (10.8)	11 (12.0)	115 (9.3)	
July	35 (10.4)	63 (9.4)	16 (11.5)	9 (9.8)	123 (10.0)	
August	42 (12.5)	70 (10.5)	10 (7.2)	8 (8.7)	130 (10.5)	
September	26 (7.7)	54 (8.1)	17 (12.2)	8 (8.7)	105 (8.5)	
October	28 (8.3)	47 (7.0)	19 (13.7)	8 (8.7)	102 (8.3)	
November	25 (7.4)	65 (9.7)	6 (4.3)	11 (12.0)	107 (8.7)	
December	19 (5.7)	43 (6.4)	9 (6.5)	4 (4.3)	75 (6.1)	
**Season**						0.338
Winter	68 (20.2)	157 (23.5)	26 (18.7)	14 (15.2)	265 (21.5)	
Spring	51 (15.2)	112 (16.8)	20 (14.4)	12 (13)	195 (15.8)	
Summer	138 (41.1)	233 (34.9)	51 (36.7)	39 (42.4)	461 (37.3)	
Autumn	79 (23.5)	166 (24.9)	42 (30.2)	27 (29.3)	314 (25.4)	
**Day of week**						0.400
Workdays	239 (71.1)	471 (70.5)	105 (75.5)	60 (65.2)	875 (70.9)	
Weekend	97 (28.9)	197 (29.5)	34 (24.5)	32 (34.8)	360 (29.1)	
**Vision 2030**						
Before	241 (71.7)	458 (68.6)	91 (65.5)	62 (67.4)	852 (69)	0.542
After	95 (28.3)	210 (31.4)	48 (34.5)	30 (32.6)	383 (31)	

**Table 4:** Patterns of head injuries and its outcome by age (*n* = 1235).

Variable	0–17 Years *n* (%)	18–34 Years *n* (%)	35–59 Years *n* (%)	≥60 Years *n* (%)	Total *n* (%)	*p* Value
**Head injury**						<0.001
Multiple lesions	115 (34.2)	213 (31.9)	43 (30.9)	26 (28.3)	397 (32.1)	
Concussion	30 (8.9)	64 (9.6)	10 (7.2)	4 (4.3)	108 (8.7)	
Contusion	41 (12.2)	96 (14.4)	12 (8.6)	11 (12)	160 (13)	
SDH * Standard residual	20 (6.0) −2.1	56 (8.4)	13 (9.4)	29 (31.5) 6.8	118 (9.6)	
EDH * Standard residual	28 (8.3)	67 (10)	11 (7.9)	2 (2.2) −2.1	108 (8.7)	
SAH * Standard residual	4 (1.2) 2.2	16 (2.4)	15 (10.8) −2	6 (6.5)	41 (3.3)	
Skull Fracture * Standard residual	51 (15.2) −2.1	72 (10.8)	11 (7.9)	4 (4.3) 4.8	138 (11.2)	
Superficial injury	14 (4.2)	18 (2.7)	8 (5.8)	2 (2.2)	42 (3.4)	
Unspecified injury	24 (7.1)	45 (6.7)	12 (8.6)	6 (6.5)	87 (7)	
Other (DAI, IVH, ICH)	9 (2.7)	21 (3.1)	4 (2.9)	2 (2.2)	36 (2.9)	
**Associated injury**						
No	174 (51.8)	302 (45.2)	59 (42.4)	50 (54.3)	585 (47.4)	0.071
Yes	162 (48.2)	366 (54.8)	80 (57.6)	42 (45.7)	650 (52.6)	
**ICU admission**						
No	276 (82.1)	537 (80.4)	109 (78.4)	80 (87)	1002 (811)	0.366
Yes	60 (17.9)	131 (19.6)	30 (21.6)	12 (13)	233 (18.9)	
**LOS**						0.124
Up to 1 week	133 (39.6)	259 (38.8)	51 (36.7)	34 (37)	477 (38.6)	
1–2 weeks	101 (30.1)	162 (24.3)	29 (20.9)	25 (27.2)	317 (25.7)	
15–30 days	53 (15.8)	112 (16.8)	29 (20.9)	13 (14.1)	207 (16.8)	
30–180 days	46 (13.7)	118 (17.7)	29 (20.9)	20 (21.7)	213 (17.2)	
>180 days	3 (0.9)	17 (2.5)	1 (0.7)	0 (0)	21 (1.7)	
**Outcome**						0.416
Discharge to home	278 (82.7)	539 (80.7)	109 (78.4)	79 (85.9)	1005 (81.4)	
Transfer	14 (4.2)	30 (4.5)	03 (2.2)	02 (2.2)	49 (4.0)	
Death	44 (13.1)	99 (14.8)	27 (19.4)	11 (12)	181 (14.7)	

*: Denotes that significant difference exist. EDH: epidural hematoma. SDH: subdural hematoma. SAH: subarachnoid hemorrhage. ICH: intracerebral hemorrhage. DAI: diffuse axonal injury. IVH: intraventricular hemorrhage. ICU: intensive care unit. LOS: length of stay.

## Data Availability

Data are available with the author and on sufficient need can be shared.
